# Prediction of Post-Infectious Inflammatory Response Syndrome in Patients with Cryptococcal Meningitis Based on Clinical and Radiomics Data

**DOI:** 10.3390/diagnostics16152370

**Published:** 2026-07-28

**Authors:** Abdilahi Abdi Ibrahim, Xiaomeng Ma, Jia Liu, Fuhua Peng

**Affiliations:** Department of Neurology, The Third Affiliated Hospital of Sun Yat-sen University, Guangzhou 510630, China; abdilahi8881@outlook.com (A.A.I.); mxiaom@mail.sysu.edu.cn (X.M.); liuj378@mail.sysu.edu.cn (J.L.)

**Keywords:** infection, inflammation, prediction, cryptococcal meningitis, radiomics

## Abstract

**Background/Objectives**: Post-infectious inflammatory response syndrome (PIIRS) is a rare complication of cryptococcal meningitis. The objective of this study was to identify predictors that can be used to identify patients at risk of PIIRS before its onset. **Methods**: A total of 149 patients with cryptococcal meningitis who did not develop PIIRS (controls, *n* = 84) and developed PIIRS (cases, *n* = 65) were included in the study. Clinical presentation data, treatment data, and laboratory data at admission were collected and compared between the two groups using univariate and multivariable analyses. Inflamed areas within T2 FLAIR MRI scans were manually annotated, and a total of 110 radiomic features were extracted per patient. Data were split into training, test, and validation sets, and multiple machine learning models integrating radiomic, spatial, and clinical features were developed and evaluated using area under the curve (AUC) and related performance metrics. **Results**: Ventriculoperitoneal shunt (odds ratio: 4.64 [1.84–12.46]; *p*-value = 0.001), complement component 3 (C3) (odds ratio: 5.78 [1.55–24.42]; *p*-value = 0.012), and lumbar puncture opening pressure (odds ratio: 1.01 [1.00–1.01]; *p*-value = 0.008) were independent risk factors associated with PIIRS development. All four radiomics models performed very well in predicting PIIRS, with the final model (region of interest + location + C3) achieving the best overall performance set (test set AUC: 0.948 [0.840–1.000], validation set AUC: 0.943 [0.814–1.000]). **Conclusions**: Radiomics-based classification models demonstrated good performance for predicting PIIRS. These findings should be considered hypothesis-generating given the small sample size and require validation in larger multicenter studies before clinical implementation.

## 1. Introduction

Cryptococcal meningitis (CM) is a serious fungal infection of the central nervous system caused primarily by *Cryptococcus neoformans* and *Cryptococcus gattii*. Though historically associated with HIV/AIDS, CM also affects patients with other forms of immunosuppression, including those with autoimmune diseases, malignancies, organ transplants, or use of immunosuppressive medications [1,2]. A substantial proportion of cases, however, occur in individuals without known immune deficits. In regions with better access to healthcare, non-HIV-related CM has become a leading cause of fungal meningitis, potentially due to wider use of immunosuppressants [2,3,4]. Diagnostic delays are common, especially in non-immunocompromised patients, as symptoms like headache and fatigue may be mistaken for less serious conditions or more prevalent infectious etiologies, including viral or bacterial meningitis [5,6]. In addition, conventional cerebrospinal fluid (CSF) diagnostic methods have limitations. India ink microscopy demonstrates variable sensitivity, particularly in patients with a low fungal burden, while fungal culture, although considered the reference standard, typically requires 7–14 days or longer for organism growth, potentially delaying diagnosis and initiation of appropriate antifungal therapy [7]. Neuroimaging, particularly magnetic resonance imaging (MRI), is an imaging modality with a high sensitivity for detecting CM-related abnormalities. Lumbar puncture remains essential for diagnosis and management, with antigen detection by lateral flow assay now the preferred method due to high sensitivity. Initial treatment includes antifungal therapy with amphotericin B and flucytosine, followed by fluconazole [8].

Despite microbiological clearance, some patients experience clinical worsening due to a post-infectious inflammatory response syndrome (PIIRS). This syndrome resembles immune reconstitution inflammatory syndrome seen in HIV patients but occurs without immune recovery [9]. PIIRS is characterized by neurological symptoms, including but not limited to cognitive decline, vision or hearing loss, and gait disturbance, despite negative fungal cultures. Inflammation is driven by persistent immune activation, including T-cell and monocyte recruitment to the central nervous system. Standard antifungal regimens are ineffective in addressing these inflammation sequalae. Thus, timely identification and treatment of PIIRS may require adjunctive anti-inflammatory approaches and careful neurologic monitoring [10].

PIIRS presents a diagnostic challenge not just for neurologists. Accurate diagnosis depends on confirming effective fungal control and identifying ongoing inflammation in the central nervous system (CNS). First, Cryptococcus infection must be ruled out: negative CSF fungal cultures are required before considering PIIRS. However, because cultures may miss residual organisms, oral antifungal therapy should continue if immunomodulation is planned. Routine CSF analyses assess the level of CNS inflammation, although much of the response occurs in the brain. Flow cytometry can quantify activated T cells in fresh CSF, and measuring cytokines (e.g., IL-6) provides additional confirmation of immune activation. Serum markers like C-reactive protein (CRP) or D-dimer are less useful to assess the severity of inflammation in the CNS but may detect other infections or thromboses. Before starting immunotherapy, clinicians should exclude other causes of meningoencephalitis by multiplex CSF panels or specific viral testing and screen for conditions that increase corticosteroid risks, such as viral hepatitis [11].

Diagnostic challenge also exists for radiologists as they must recognize that MRI findings may be due to PIIRS and not treatment failure. Limited visual interpretation (caused by inter-rater variability, time-consuming nature of manual assessment, etc.) [12,13,14,15] further affects diagnostic utility of MRI in detecting PIIRS. The integration of artificial intelligence, specifically machine learning, has revolutionized medical imaging by enabling the extraction of quantitative features (radiomics) [16,17,18]. However, the application of radiomics tools to clinical data is difficult. The vast majority of neuroimaging analysis software is optimized for high-resolution T1/T2-weighted scans and less developed for other important MRI sequences such as FLAIR [19]. FLAIR sequences provide high lesion-to-tissue contrast, making them particularly effective for detecting inflamed regions [20,21,22]. Studies showed that contrast-enhanced FLAIR can be more sensitive than T1-weighted imaging in certain meningeal conditions [23,24,25]. Given the nature of PIIRS, neither imaging nor clinical variables alone are likely to fully characterize disease risk. Therefore, integrating quantitative FLAIR-derived radiomic features with relevant clinical biomarkers may provide crucial information for risk stratification and improve prediction of PIIRS development [26]. There is currently a lack of integrated models that combine quantitative FLAIR-derived radiomic features with relevant clinical and laboratory biomarkers to predict PIIRS before its clinical onset. Existing research has largely avoided this area of research likely due to the rarity of PIIRS and overall complexity. Accordingly, this study was designed to address this gap by developing and evaluating multimodal machine learning models that integrate FLAIR-based radiomic features, spatial imaging descriptors, and clinical biomarkers to predict PIIRS in non-HIV patients with CM.

## 2. Materials and Methods

### 2.1. Study Design and Population

This retrospective cohort study was conducted at the Third Affiliated Hospital of Sun Yat-sen University, spanning January 2012 through March 2025. Patients were included if they were above 18 years old, had a confirmed CM diagnosis and, for those developing PIIRS, at least one week of available clinical data before PIIRS onset. Exclusion criteria were pregnancy, neoplasm, HIV infection, or any patient lacking sufficient data at least one week before PIIRS onset. The patients were included irrespective of the use of immunosuppressive agents. All patients were treated in general wards. Patients were temporarily transferred to the intensive care unit (ICU) when their lives were in critical condition. The definition of PIIRS was as follows: In a previously healthy patient whose CSF cultures converted to negative after amphotericin-based induction, the syndrome was characterized by new or worsening neurological dysfunction: persistent or declining cognition, non-refractive visual deficits, or auditory changes. Supportive evidence included CSF biomarkers (increased white blood cells and protein, decreased glucose levels), elevated inflammatory markers in CSF (IL-6, soluble CD25), increased levels of activated immune cells (HLA-DR^+^ CD4^+^ T cells, HLA-DR^+^ CD8^+^ T cells, natural killer cells, monocytes), and characteristic MRI abnormalities on post-contrast FLAIR sequences [11].

### 2.2. Data Collection and Definitions

We extracted demographic, clinical, radiological, laboratory, and treatment variables from electronic medical records. Key baseline characteristics included age, sex, various dates (symptom onset, first treatment, PIIRS onset, etc.), and initial neurologic symptoms (e.g., headache, vision or hearing loss). Treatment data comprised pre- and post-admission use of antifungals (flucytosine, amphotericin B formulations, fluconazole, voriconazole) and corticosteroids, as well as ventriculoperitoneal shunt (VPS) placement. Laboratory data markers (e.g., white blood cell count, complement component (C3)) were recorded. All imaging was axial T2 FLAIR MRI scans obtained prior to PIIRS onset. Each examination contained 19 sequential slices.

### 2.3. Image Annotation Processing and Binary Mask Generation

**Figure 1:**
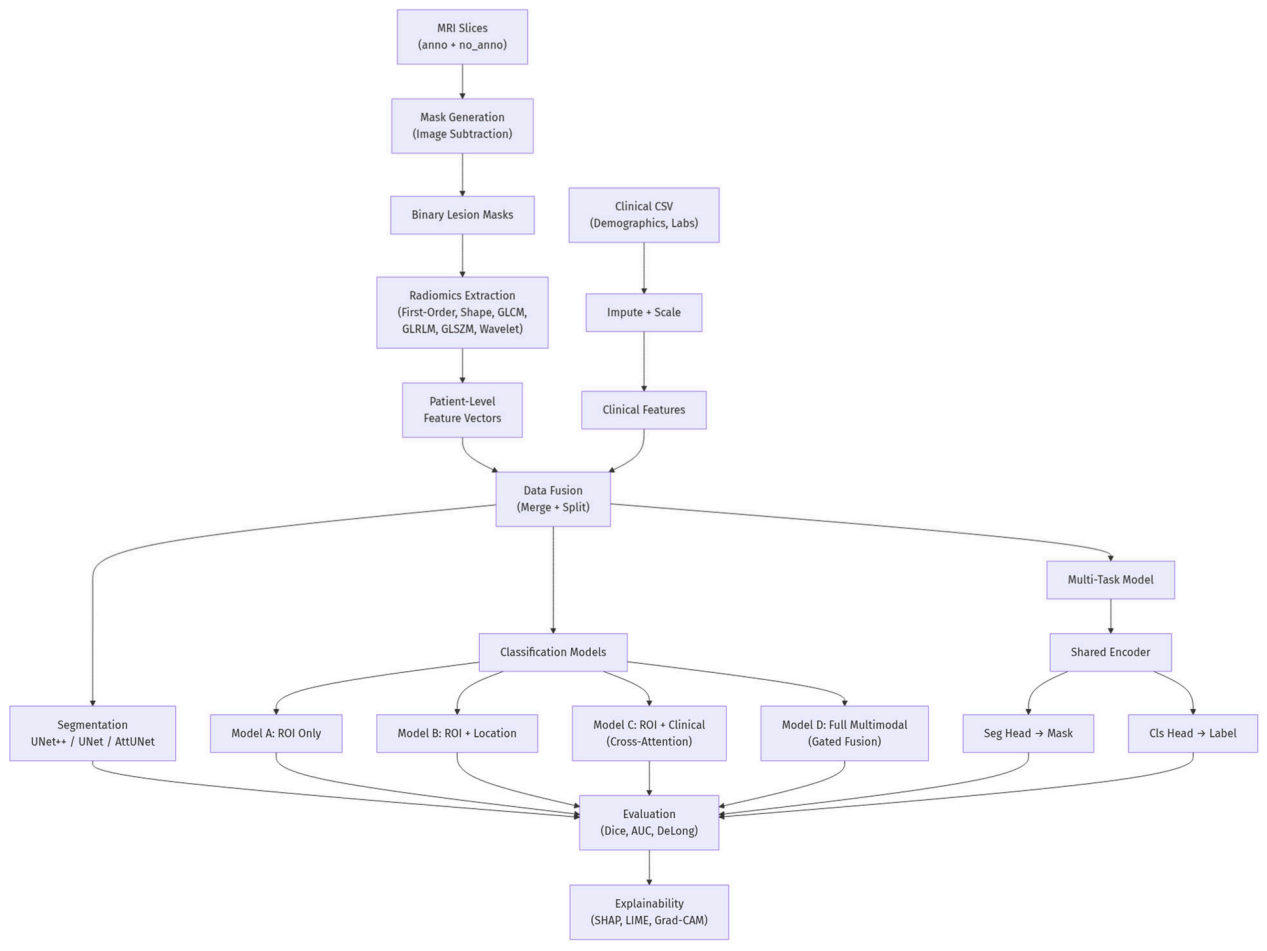
Flowchart of the radiomics study. GLCM—gray-level co-occurrence matrix, GLRM—gray-level run length matrix, GLSZM—gray-level size zone matrix, ROI—region of interest, AUC—area under the curve, SHAP—SHapley Additive exPlanations, LIME—Local Interpretable Model-agnostic Explanations.

Machine learning analysis was conducted in Python (v.3.14.0) with PyTorch (v.2.9.1) as the deep-learning framework and modularized into preprocessing, mask generation, radiomics extraction, fusion, segmentation, classification, multi-task training, evaluation, explainability and visualization steps (flowchart is shown in Figure 1). Key libraries included OpenCV (v.4.13.0.92) for image processing, scikit-learn (1.8.0) for splitting and preprocessing, scikit-image (v.0.26.0) and SciPy (1.17.0) for texture and statistical computations, SHAP (v.0.50.) and LIME (v.0.2.0.1) for interpretability, and various libraries (specifically, OpenCV (v.4.13.0.92), scikit-image (v.0.26.0), NumPy (v.2.4.2), PyWavelets (v.1.9.0)) for feature extraction.

Each axial T2 FLAIR scan was manually annotated by selecting lesions within the brain using a 3D slicer. Manual annotation was performed by two neurologists independently; annotations were then compared and any differences were eliminated following discussion. Binary lesion masks were produced automatically by comparing annotated images with their corresponding non-annotated originals. Three detection strategies were implemented and evaluated: (1) pixel subtraction, where the absolute per-pixel difference between the annotated and original grayscale images was computed, augmented by the maximum per-channel color difference, smoothed with a 5 by 5 Gaussian kernel and thresholded at 25 to produce a binary map; (2) color-space thresholding, where the annotated image was converted to HSV color space, high-saturation regions corresponding to colored overlays were detected and combined with a color difference map versus the original image; (3) a combined method that used the union of the subtraction and color-threshold masks. In all methods morphological closing (two iterations) followed by opening (one iteration) with a 3 by 3 elliptical kernel was applied to clean the masks, contours smaller than 50 pixels were discarded, and final masks were resized to 256 by 256 pixels using nearest-neighbor interpolation and saved as binary PNG files. Slices without detectable overlays were labeled non-lesion slices and retained to preserve anatomical context. The generated masks were used for segmentation model training and for slice-level radiomics extraction.

### 2.4. Lesion Localization and Segmentation Models

Lesion localization was performed using a UNet++ (Nested U-Net) architecture with deep supervision. The network consisted of five encoder levels with feature dimensions [32, 64, 128, 256, 512], densely connected skip pathways that aggregate features from all preceding nodes at each resolution level, and multiple output heads at each decoder level. During inference, the deepest supervision output was used. All segmentation architectures used double 3 × 3 convolution blocks with batch normalization and rectified linear unit (ReLU) activations. Additionally, Squeeze-and-Excitation blocks were implemented for optional channel attention.

The segmentation model was trained using a combined loss function consisting of a weighted sum of Dice loss (weight = 0.7) and binary cross-entropy loss (weight = 0.3). The Adaptive Moment Estimation with Weight Decay (AdamW) optimizer was used with an initial learning rate of 1 × 10^−4^ and weight decay of 1 × 10^−5^. A cosine annealing learning rate schedule was applied. Training ran for up to 10 epochs with early stopping (patience = 10 epochs, monitoring validation loss). Gradient norms were clipped to a maximum of 1.0. Data augmentation during training included random horizontal flips, vertical flips, random rotations (±15°), random brightness/contrast adjustments, and additive Gaussian noise (σ = 0.02). Predicted masks were evaluated against reference masks using the Dice similarity coefficient, Intersection over Union (IoU), precision, and recall.

### 2.5. Radiomics Feature Extraction and Aggregation

In total, 110 radiomics features were extracted per patient. Radiomic features were computed on segmented lesion regions on a per-slice basis and aggregated to the patient level by mean pooling across lesion-containing slices. Feature categories were as follows: (1) first-order statistics (20 features), (2) shape (13 features), gray-level co-occurrence matrix (GLCM) (18 features), (3) gray-level run length matrix (GLRLM) (11 features), (4) gray-level size zone matrix (GLSZM) (11 features), (5) neighborhood gray-tone difference matrix (NGTDM) (5 features), (6) wavelet features (32 features).

Spatial features were aggregated at the patient level from slices containing lesion region of interest (ROIs; abnormal signals on T2 FLAIR MRI) and encoded as a compact descriptor of three-dimensional extent and distribution: (1) the number of ROI-containing slices, (2) the fraction of slices with ROI, (3) the mean and standard deviation of the normalized slice position, (4) the minimum and maximum lesion slice indices, (5) the mean lesion centroid coordinates (x, y), (6) the total, mean, and maximum ROI area.

### 2.6. Multimodal Model Development

Data were divided into training (68%), validation (12%), and independent test (20%) sets using stratified random splitting to maintain class balance and prevent patient overlap across splits. Preprocessing for tabular features (imputation and scaling) was then performed in a leakage-safe manner: parameters were fit using training data only and subsequently applied unchanged to validation and test sets. The same patient-level split was used consistently across all model training and evaluation. After merging radiomics, spatial, and clinical features into a patient-level matrix, infinite values were replaced with NaN and subsequently handled during preprocessing. Feature normalization (z-score StandardScaler) was performed after train/validation/test splitting: the scaler was fit on the training subset only and then applied to validation and test subsets using the same fitted parameters. This ensured comparable feature scales while avoiding information leakage from non-training data. In addition, we performed stratified patient-level 3-fold cross-validation using the same preprocessing and modeling. Four classification models (models A–D) were developed using multi-layer perceptron (MLP) architectures as well as a multi-task deep learning model:

1. Model A (ROI only): an MLP classifier receiving radiomics features alone. The network architecture consisted of two hidden layers with dimensions [64, 32], each followed by batch normalization, ReLU activation, and dropout (*p* = 0.3). A final linear layer produced a single scalar logit for binary classification.
2. Model B (ROI + location): a dual-branch architecture in which the radiomics branch projected features to 48 dimensions and the spatial location branch projected to 16 dimensions, each via a linear layer with batch normalization, ReLU, and dropout. The concatenated 64-dimensional representation was then processed by the shared MLP classifier backbone (hidden layers [64, 32]).
3. Model C (ROI + clinical data): A dual-branch architecture incorporating cross-attention fusion. Both the radiomics and clinical branches projected to a shared embedding dimension of 64. Multi-head attention (4 heads) was applied, in which the radiomics representation attended to the clinical feature representation. The attention output was concatenated with the clinical embedding to form a 128-dimensional fused representation, which was then processed by the shared MLP classifier backbone.
4. Model D (ROI + location + clinical data—full multimodal fusion): a three-branch architecture with gated fusion. Each branch (radiomics, location, clinical data) independently projected its inputs through two fully connected layers to a 64-dimensional embedding (the location branch used an intermediate 32-dimensional projection). The three embeddings were concatenated into a 192-dimensional vector, passed through a sigmoid gate that learned modality-specific importance weights, and element-wise multiplied with the concatenation before classification by the shared MLP backbone.
5. Multi-Task Model (joint segmentation + classification): a shared convolutional encoder with feature dimensions [32, 64, 128, 256] and a double 3 × 3 convolution bottleneck (512 channels) fed into two task-specific heads: (1) a segmentation decoder that mirrored the encoder with transposed convolutions and skip connections to produce a pixel-level lesion mask; and (2) a classification head that applied global average pooling to the bottleneck features, optionally concatenated clinical features, and passed the result through a two-layer MLP (256 -> 128 -> 1) with batch normalization and dropout (*p* = 0.3). Slice-level classification logits were mean-pooled across all slices within a patient to produce a PIIRS prediction. To manage GPU memory, slices were processed in chunks of 4 during training.

### 2.7. Loss Functions and Training

All classification-only models were trained with binary cross-entropy with logits loss, optionally applying class weights to compensate for label imbalance. The multi-task model optimized a combined objective written as L_total = w_seg · L_seg + w_cls · L_cls, where L_seg was the weighted Dice plus binary cross-entropy segmentation loss and L_cls was binary cross entropy (BCE)-with-logits classification loss, with w_seg = w_cls = 0.5. Classification models (A-D) were optimized with AdamW (learning rate = 5 × 10^−4^, weight decay = 1 × 10^−4^) and a cosine annealing learning rate schedule (minimum learning rate = 5 × 10^−6^) for up to 500 epochs with early stopping. Dropout regularization (*p* = 0.3) was applied throughout all hidden layers. During training, additive Gaussian noise (σ = 0.02) was applied to input feature vectors as a data augmentation strategy. The multi-task and segmentation models used AdamW (learning rate = 1 × 10^−4^, weight decay = 1 × 10^−5^) with cosine annealing (minimum learning rate = 1 × 10^−6^) for up to 50 epochs with early stopping. All models used gradient clipping (max norm = 1.0).

### 2.8. Model Evaluation Model Interpretation

Classification performance was assessed primarily by AUC score with bootstrapped 95% confidence intervals (1000 iterations) and secondarily by accuracy, sensitivity, specificity, positive predictive value, negative predictive value, and F1 score. Thresholds were selected using Youden’s J statistic. Segmentation was evaluated by Dice coefficient, intersection over union, precision and recall, reported as mean ± SD across slices that contained ground-truth ROIs. Decision curve analysis across threshold probabilities from 0.01 to 0.99 was used to estimate net benefit against treat-all and treat-none strategies. Pairwise comparisons of correlated AUCs between classification models were performed using the DeLong test with significance at *p* < 0.05.

Model explanations combined complementary approaches for tabular and image models: (1) permutation-based feature importance, computed with ten repetitions of feature permutation and using AUC degradation as the metric; (2) SHapley Additive exPlanations (SHAP) analysis with KernelExplainer for tabular classifiers using a background set of up to 50 instances and beeswarm plots summarizing the top 20 features; (3) Grad-CAM and Grad-CAM++ for convolutional models targeting the deepest convolutional layer, where gradient-weighted activation maps were upsampled to input resolution by bilinear interpolation and ReLU-activated to highlight positively contributing regions; and (4) Local Interpretable Model-agnostic Explanations (LIME) for per-image local explanations of the segmentation/classification outputs using superpixel perturbations (1000 perturbed samples per instance and 20 features) to generate positive-only and combined region importance maps.

### 2.9. Statistical Analysis

All statistical analyses were performed using R (4.4.1). Continuous variables were tested for normality by the Shapiro–Wilk test. Normally distributed data are presented as mean ± SD and compared by Student’s *t*-test, and non-normal data as median [first quantile—third quantile] with Mann–Whitney U test. Categorical variables are reported as counts (percentages) and compared using the Chi-squared test or Fisher’s exact test. A *p*-value adjustment was not carried out. Variables with *p* < 0.05 in univariate analyses were entered into multivariable logistic regression to identify independent risk factors associated with PIIRS development. Unadjusted two-sided *p* < 0.05 was considered statistically significant.

## 3. Results

### 3.1. Clinical Presentation

A total of 149 patients with CM were divided into a control group (*n* = 84) and a PIIRS group (*n* = 65) based on subsequent development of PIIRS. There were no significant differences in terms of demographic and temporal variables: the mean age was 47.87 ± 16.11 years in controls vs. 45.49 ± 15.66 years in PIIRS patients (*p* = 0.368), and the interval from symptom onset to initiation of treatment was 20.00 [13.00–32.00] days in controls compared with 26.00 [14.50–46.00] days in the PIIRS group (*p* = 0.142) (Table 1). Gender distribution showed no significant difference, with males comprising 61.9% of controls and 73.8% of PIIRS cases (*p* = 0.124). Headache occurrence was similar in both groups (95.2% vs. 95.4%, *p* = 1.0). Also, fever (65.1% vs. 53.8%, *p* = 0.167) and nausea (60.2% vs. 72.3%, *p* = 0.125) did not differ significantly. In contrast, several neurological manifestations were significantly more common in the PIIRS group. Vision loss occurred in 50.8% of PIIRS patients vs. 26.5% of controls (*p* = 0.002), and hearing loss affected 27.7% of the former compared with 12.2% of the latter (*p* = 0.017). Vomiting was also significantly more frequent in PIIRS cases (72.3% vs. 51.8%, *p* = 0.011). Interestingly, limb paralysis was observed in 8.4% of controls but in none of the PIIRS patients (*p* = 0.019). Other neurological signs, such as consciousness disturbance, epilepsy, and psychiatric symptoms, exhibited no statistically significant differences between the two groups. Finally, immunosuppressant usage was significantly higher in the control group than in the PIIRS group (19.3% vs. 4.6%, *p* = 0.008).

### 3.2. Treatment Regimen

All patients were treated in general wards. A total of 63 patients were temporarily transferred to the ICU, all of whom underwent VPS placement (21 patients and 42 patients in the control and PIIRS groups, respectively). After passing the critical period, these patients were transferred back to the general wards. Antifungal therapy before admission was similar between groups: flucytosine (16.9% vs. 19.3%, *p* = 0.726), amphotericin B (33.8% vs. 33.3%, *p* = 0.955), fluconazole (21.1% vs. 26.3%, *p* = 0.491), voriconazole (5.6% vs. 1.8%, *p* = 0.380), and glucocorticoids (4.3% vs. 5.3%, *p* = 0.796). After admission, PIIRS patients had a significantly lower overall glucocorticoid exposure (28.1% vs. 46.9%, *p* = 0.021), but significantly more often received amphotericin B (90.5% vs. 75.0%, *p* = 0.017). Notably, VPS placement was significantly more frequent among those who developed PIIRS (64.6% vs. 25.0% in controls, *p* < 0.001). The timing of VPS insertion after symptom onset was similar between groups at median 48–49 days (Table 2).

### 3.3. Laboratory Data

Laboratory data are provided in Table 3. C3 was significantly elevated in PIIRS patients (1.26 ± 0.36 g/L) compared with controls (1.08 ± 0.39 g/L, *p* = 0.005), while C4 was also higher in PIIRS patients but the difference was not statistically significant (0.31 [0.25–0.43] g/L vs. 0.29 [0.21–0.38] g/L, *p* = 0.107). Significantly higher levels of CRP were observed in PIIRS patients: 8.80 [2.50–25.00] mg/L vs. 4.65 [1.45–13.80] mg/L in controls (*p* = 0.043). However, procalcitonin levels did not significantly differ between the two groups (0.09 [0.05–0.25] vs. 4.72 [3.83–5.20] ng/mL, *p* = 0.374). Notably, opening pressure on lumbar puncture was significantly higher in those who developed PIIRS (275.00 [170.00–330.00] mmH_2_O) than in the controls (197.50 [154.00–275.00] mmH_2_O), *p* = 0.008. Finally, quantitative CSF cryptococcal smear burden was significantly higher in PIIRS patients with a median of 5376.00 [514.00–23,168.00] vs. 469.50 [10.00–4154.00] in the controls (*p* < 0.001), whereas CSF chloride was significantly higher in the control group than in the PIIRS group (117.85 (113.00–122.50) vs. 115.70 (109.00–118.90, *p* = 0.012).

### 3.4. Multivariable Analysis

Risk factors obtained from the univariate analysis were used for multivariable analysis (Table 4). In a logistic regression model adjusted for key clinical, radiological, and laboratory variables, three independent predictors of PIIRS emerged. VPS placement was associated with a more than threefold higher odds of developing PIIRS (OR 4.64, 95% CI 1.84–12.46; *p* = 0.001). Moreover, serum C3 and lumbar puncture opening pressure were independent risk factors associated with PIIRS development (OR 5.78, 95% CI 1.55–24.42; *p* = 0.012 and OR 1.01, 95% CI 1.00–1.01; *p* = 0.008, respectively).

### 3.5. Radiomics Analysis

Among 149 patients included in the study, 122 patients had T2 FLAIR MRI scans (53 PIIRS cases and 69 controls). A total of 110 radiomic features were extracted per patient. Several variables were significantly different between the two groups, including mean signal intensity and total ROI (Supplementary Tables S1–S7).

**Figure 2:**
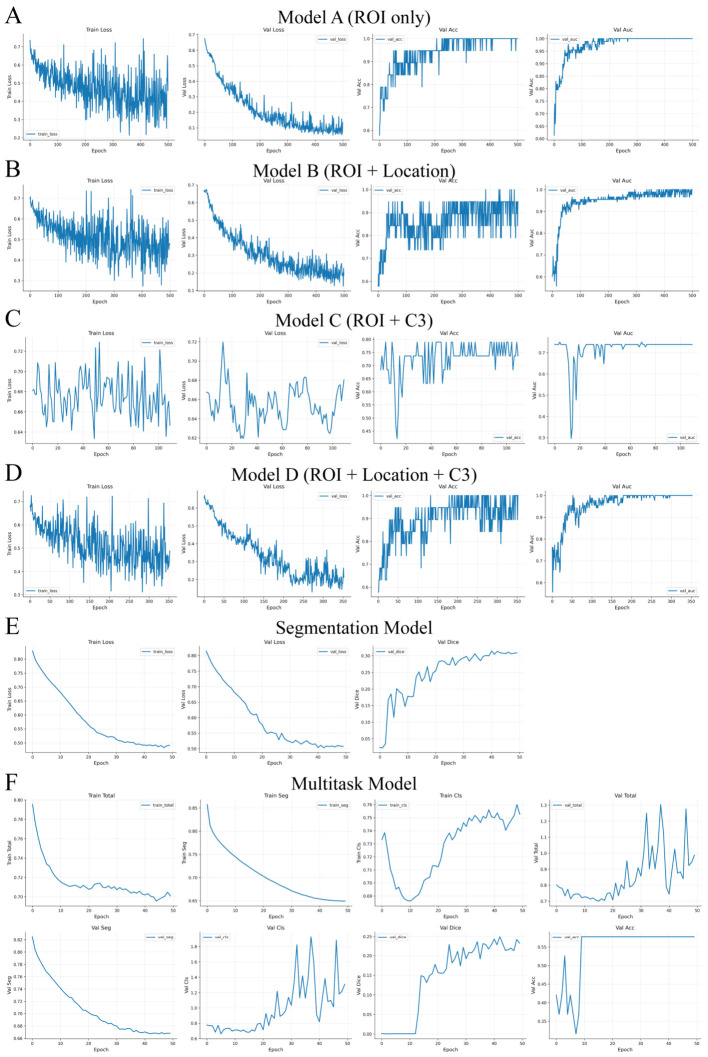
Training curves for models showing training loss, validation loss, validation accuracy, and validation AUC over epochs. (**A**) ROI only. (**B**) ROI + location. (**C**) ROI + C3. (**D**) ROI + location + C3. (**E**) Segmentation model. (**F**) Multitask model.

Figure 2 shows model convergence, stability, and generalization performance during training. Overall, models A, B, and D demonstrated strong and stable convergence with high AUC. In contrast, validation performance for model C was suboptimal and unstable, with AUC plateauing around 0.74. The segmentation model was also associated with limited performance (both training and validation losses decreasing and validation Dice improving to ~0.30). Finally, the results for the multitask model (segmentation + classification) were poor.

**Figure 3:**
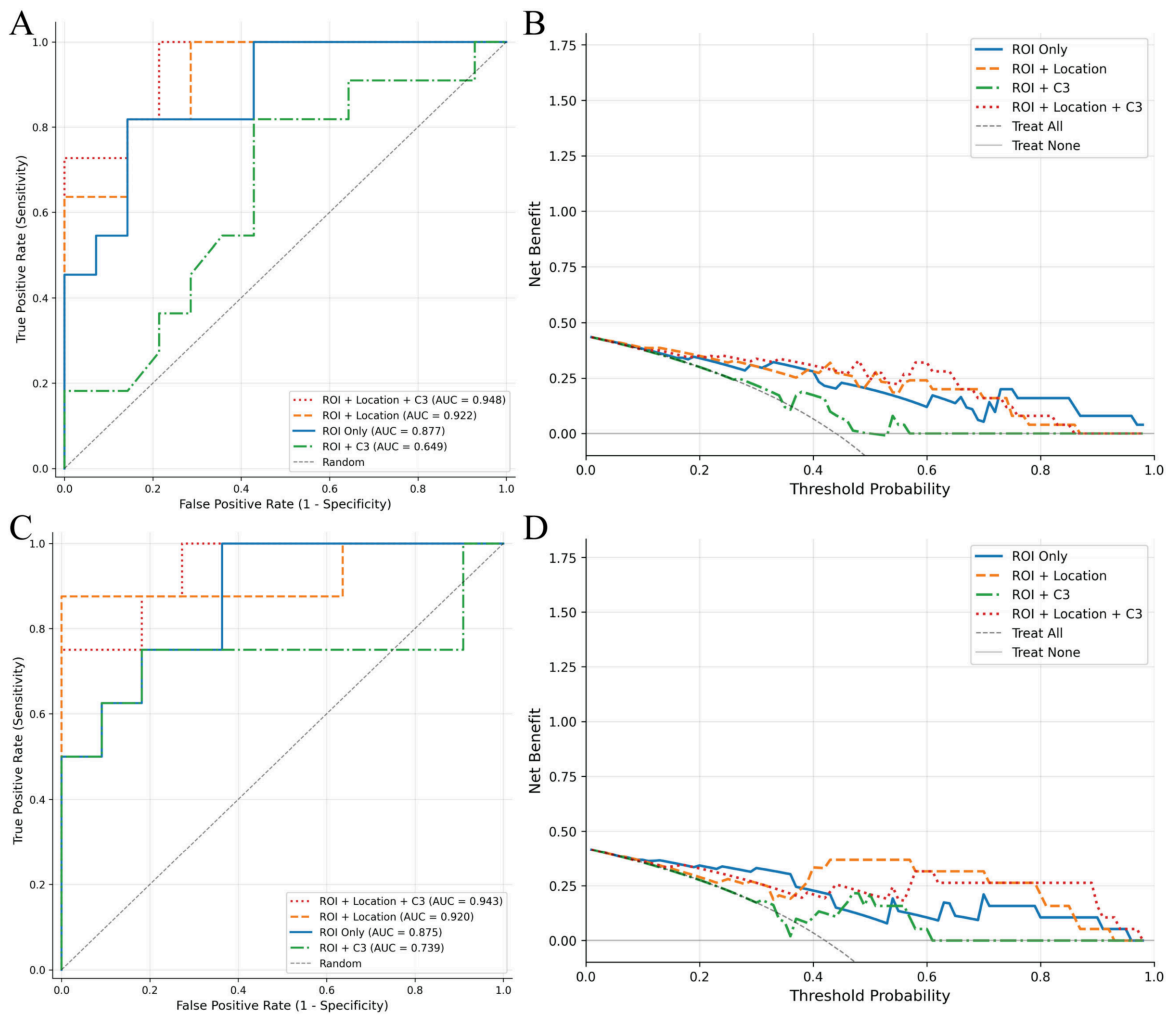
Model results. (**A**) Receiver operating characteristic curve (test set). (**B**) Decision curve (test set). (**C**) Receiver operating characteristic curve (validation set). (**D**) Decision curve (validation set).

All planned classification experiments (prediction of PIIRS) completed successfully and produced good results as evidenced by receiver operating characteristic and decision curves (Figure 3 and Table 5). The ROI-only model demonstrated good performance in both the test set (AUC: 0.877, 95% CI [0.714–0.991]) and validation set (AUC: 0.875, 95% CI [0.656–1.000]). The model B (ROI + location) showed strong performance, with AUCs of 0.822 (95% CI [0.800–1.000]) in the test set and 0.920 (95% CI [0.733–1.000]) in the validation set, achieving perfect specificity in the latter. The model C (ROI + C3) exhibited the weakest performance among all models, with lower AUCs in both the test set (AUC: 0.649, 95% CI [0.429–0.864]) and validation set (AUC: 0.739, 95% CI [0.411–0.982]). In contrast, the final model (ROI + location + C3) achieved the best overall performance, with high AUCs in both the test set (AUC: 0.948, 95% CI [0.840–1.000]) and validation set (AUC: 0.943, 95% CI [0.814–1.000]). Pairwise DeLong testing revealed that the ROI + C3 model performed significantly worse than both the ROI + location model (*p* = 0.0436) and the ROI + location + C3 model in the test set (*p* = 0.0137), while no significant differences were observed among the other model comparisons (*p* > 0.05). Following three-fold cross-validation, models had similar performance. However, pairwise DeLongi test revealed that there were no significant differences in their performance (Supplementary Figure S1).

**Figure 4:**
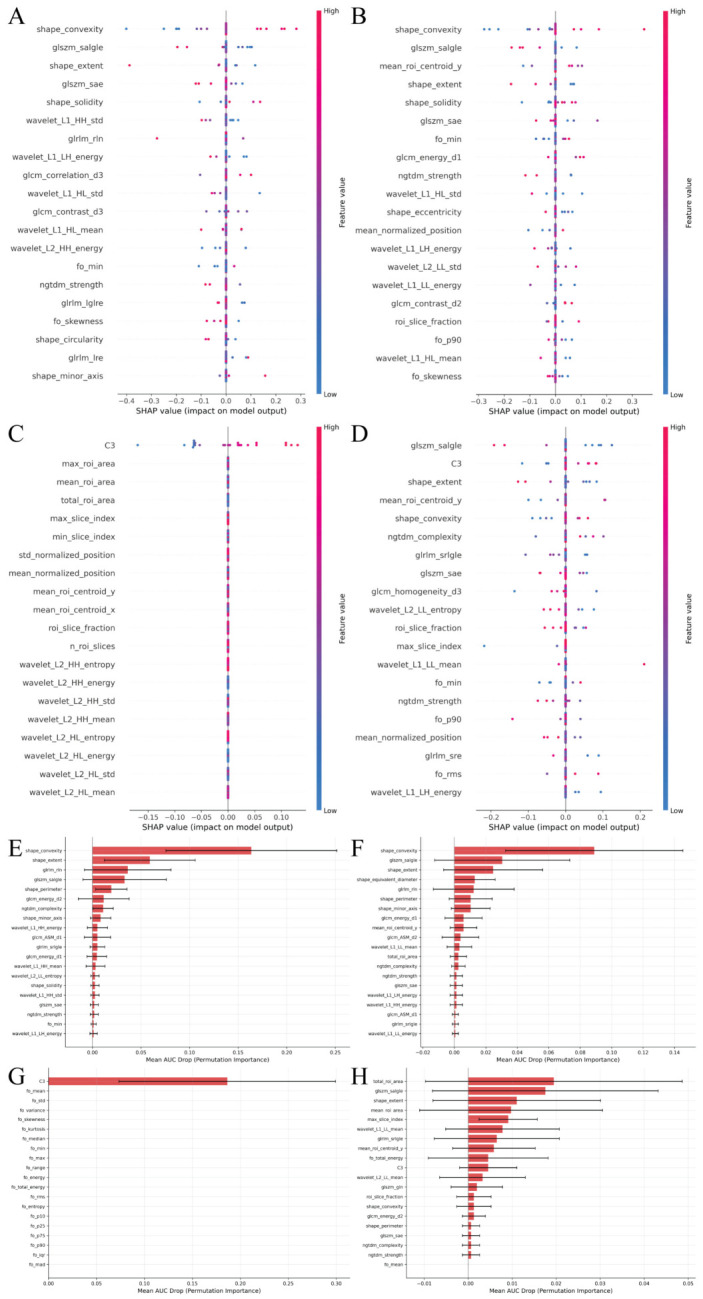
Explainable algorithms for classification models (**A**–**D**) SHAP beeswarm plot for (**A**) ROI only. (**B**) ROI + location. (**C**) ROI + C3. (**D**) ROI + location + C3. (**E**–**H**) Permutation feature importance for (**E**) ROI only. (**F**) ROI + location. (**G**) ROI + C3. (**H**) ROI + location + C3.

**Figure 5:**
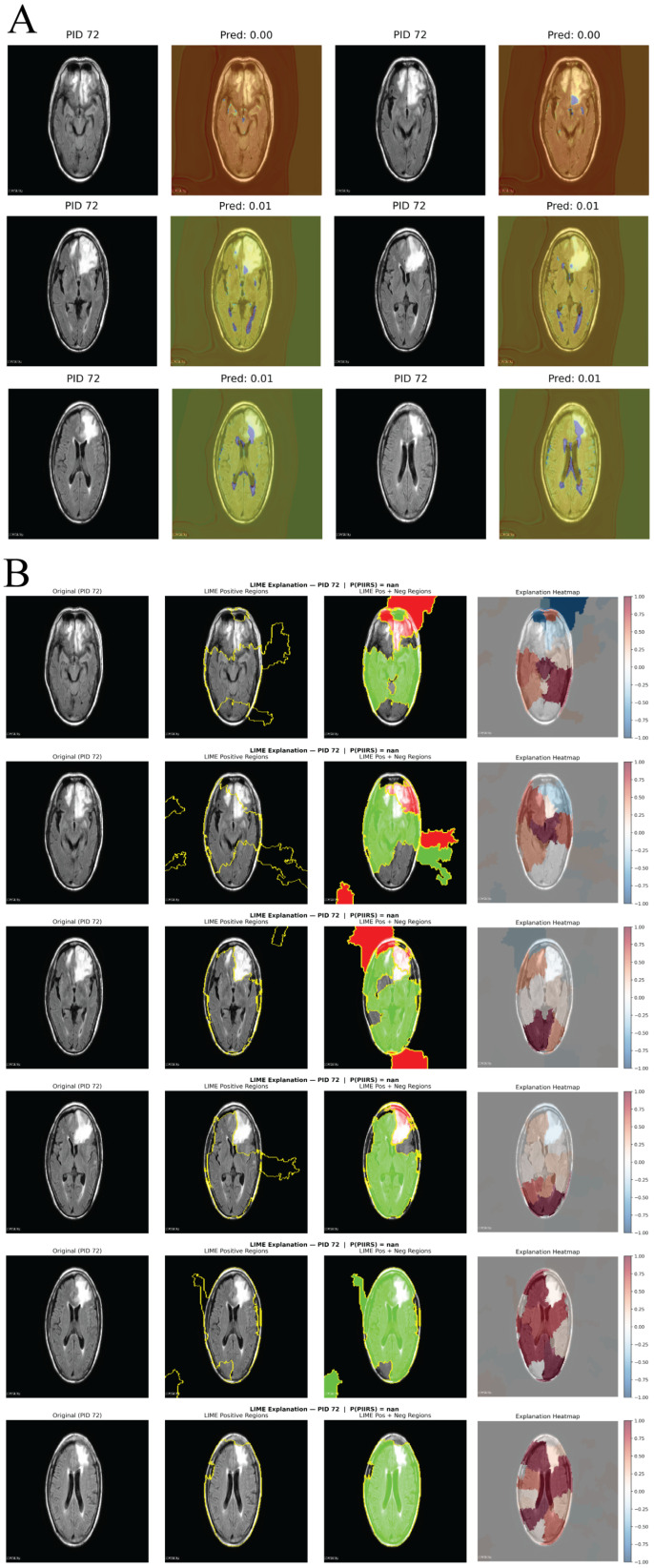
Explainable algorithms for segmentation + classification tasks. (**A**) Grid-CAM attention maps. (**B**) LIME plot. Green color supports the predicted class (post-infectious inflammatory response syndrome), whereas red color opposes it.

According to explainable algorithms (Figure 4), shape convexity and small-area low gray-level emphasis (“glszm_salgle”) had the highest impact on the performance of model A (ROI only) and model B (ROI + location). In model C, only C3 influenced the model’s decisions. Finally, ROI + location + C3 model’s performance was mostly impacted by small-area low gray-level emphasis, C3 and shape extent. Figure 5 shows model’s segmentation + classification capabilities, which were mostly nonsensical and poor overall.

## 4. Discussion

In this study, we developed and evaluated artificial intelligence-based models to predict the onset of PIIRS in patients with CM using pre-onset T2 FLAIR MRI and associated clinical data. In addition to predictive modeling, the study aimed to localize inflammatory regions in the brain using automated lesion segmentation and to evaluate the contributions of lesion morphology, spatial distribution, and clinical variables to predictive performance. A multimodal analytical method was implemented that combined medical image preprocessing, automated mask generation, radiomics feature extraction, deep learning-based segmentation and classification, and explainable algorithms.

In our cohort, PIIRS patients and controls were largely similar at baseline in terms of demographics, clinical presentation, timing of treatment, and comorbidities. The interval from symptom onset to initiation of antifungal therapy (median 20.0 vs. 26.0 days) and to laboratory analysis (median 26.0 vs. 31.0 days) did not differ significantly between controls and PIIRS cases. This similarity in timing is crucial, as it minimizes confounding by disease duration or diagnostic delay when comparing inflammatory markers and outcomes between groups. Although the comparable timing of treatment and laboratory evaluation across groups strengthens the validity of our study, it would be ideal to collect samples at standardized time points relative to symptom onset. Unfortunately, PIIRS remains too rare for such designs, and many patients are referred after initial care elsewhere, introducing unavoidable variability.

Most of the variables from the laboratory analysis of blood and CSF were not significantly different between the two groups. An earlier study identified IgM, IL-6, D-dimers, and other variables as significant predictors of PIIRS [27]. However, we did not observe significant differences between the PIIRS and non-PIIRS groups. This discrepancy may be attributed to different study designs, as we included immunocompromised individuals in our study. Multivariable analysis identified VPS placement, elevated serum C3, and higher lumbar puncture opening pressure as independent risk factors associated with PIIRS. VPS insertion was likely shown to increase PIIRS risk by indicating severe disease at baseline or even facilitating neuroinflammation [28,29,30]. Elevated lumbar puncture opening pressure, which is indicative of elevated intracranial pressure, is consistently associated with greater morbidity and mortality in fungal meningitis. Moreover, high intracranial pressure in CM is associated with poor outcome, and aggressive CSF drainage improves survival [31,32]. Animal models of bacterial meningitis demonstrate that C3 is essential for effective bacterial clearance and leukocyte recruitment. C3-deficient mice suffer higher bacterial loads and worse systemic outcomes despite reduced intracranial inflammation [33]. Moreover, in human Pneumococcal meningitis, low CSF C3 but not serum C3 was significantly associated with increased mortality [34]. A study on COVID-19 showed that C3 levels rise dramatically in severe disease, correlating with ICU admission [35]. Likewise, epidemiological data link consistently high C3 to low-grade inflammatory conditions such as metabolic syndrome [36].

A total of 110 features from six feature groups were extracted. First-order statistics quantify basic voxel intensity distributions such as mean, median, variance, and entropy. Shape descriptors describe geometric properties of segmented regions such as area, perimeter, compactness, and eccentricity [37]. GLCM features capture pairwise relationships between neighboring gray levels and report metrics like contrast and homogeneity [38]. GLRLM encode the length and gray-level composition of contiguous runs of voxels [39,40]. GLSZM features summarize the size and intensity of homogeneous zones across the lesion [41]. NGTDM characterize local perceptual properties such as busyness, coarseness and complexity [42,43,44]. Wavelet features decompose the image into frequency subbands to reveal multi-scale textural patterns [38].

Segmentation models produced “coarse” lesions, and the networks achieved modest overlap metrics (Dice ~0.30). This is typical for lesion detection on MRI scans where lesion appearance is variable. Although performance is acceptable, it is insufficient for clinical-grade segmentation. In addition, the multi-task model (joint segmentation + classification) performed very poorly, with low classification accuracy and minimal segmentation improvement. Prior work has shown that achieving high Dice scores on heterogeneous clinical FLAIR series can be difficult and often requires large, curated training sets or consensus labels [45]. Methods that transfer labels from higher quality sequences or leverage semi-supervised learning can improve results for FLAIR segmentation [46]. In addition, clinical MRI acquisitions present large variability in slice spacing, thickness, and contrast, and robust segmentation tools must tolerate that variability to be useful in practice [47].

In contrast, classification models were associated with optimal performance. The radiomics-only model (model A: ROI only) demonstrated good performance in both test and validation sets. In other words, radiomic features alone were sufficient to predict the onset of PIIRS in this study. The addition of spatial information (model B: ROI + location) further improved performance. In contrast, incorporation of C3 (model C) resulted in inferior predictive performance. Although C3 was identified as an independent risk factor associated with PIIRS in multivariable logistic regression, its integration into a deep learning model did not improve predictive performance. This discrepancy is likely caused by differences in conventional statistical analysis tools and deep learning algorithms. To put it simply, logistic regression captures linear associations and performs well in small datasets, whereas deep learning models attempt to learn complex patterns within data and perform better in large datasets. The best overall performance was achieved by the full multimodal model (model D: ROI + location + C3), which was associated with the highest AUC in both validation and test sets. Importantly, no significant differences were observed among the top-performing models (A, B, and D), whereas model C performed significantly worse in the test set.

The main limitation of our study is the rarity of PIIRS. Given how seldom it is diagnosed, it is extremely difficult to construct a large cohort. Thus, the study warrants expanded validation in larger cohorts and prospective evaluation to establish clinical value. Future work should refine segmentation with additional high-quality annotations and explore further multimodal biomarkers to improve generalizability.

## 5. Conclusions

VPS placement, elevated serum C3 levels, and increased lumbar puncture opening pressure were identified as independent risk factors associated with PIIRS. Radiomics-based classification models demonstrated overall good performance for predicting PIIRS. Segmentation and multitask models performed poorly and displayed insufficient performance for clinical application. However, the study’s results should be interpreted with caution. The small sample size, single-center retrospective design, and lack of external validation limit the clinical applicability of the constructed models. In addition, the clinical applicability of the final models is limited by the requirement for both T2 FLAIR MRI and serum C3 measurement, which may not always be available. This further limits the use of the predictive models as standalone decision-support tools. Therefore, our models should be regarded as exploratory and hypothesis-generating rather than immediately applicable in clinical practice. Additional validation in larger and preferably multicenter cohorts is required before these models can be considered for routine clinical use. Future directions may include automated lesion segmentation, longitudinal MRI changes before PIIRS onset, and evaluation of additional imaging sequences and clinical biomarkers.

## Figures and Tables

**Table 1:** Baseline characteristics and clinical presentation.

Item	Control Group (*n* = 84)	PIIRS Group (*n* = 65)	*p* Value
Gender (*n* = 149)			0.124
Male	52 (61.9%)	48 (73.8%)	
Female	32 (38.1%)	17 (26.2%)	
Age (*n* = 149)	47.87 ± 16.11	45.49 ± 15.66	0.368
From onset to treatment (d), median [Q1, Q3] (*n* = 148)	20.00 (13.00, 32.00)	26.00 (14.50, 46.00)	0.142
From onset to PIIRS (d) (*n* = 64)	-	87.67 ± 54.93	-
From onset to laboratory analysis (d), median [Q1, Q3] (*n* = 149)	26.0 [16.5, 59.0]	31.0 [22.0, 60.0]	0.202
from negative smear to PIIRS (d) (*n* = 65)	-	35.06 ± 44.00	-
Headache (*n* = 148)	79 (95.2%)	62 (95.4%)	1.0
Fever (*n* = 148)	54 (65.1%)	35 (53.8%)	0.167
Nausea (*n* = 148)	50 (60.2%)	47 (72.3%)	0.125
Vomiting (*n* = 148)	43 (51.8%)	47 (72.3%)	0.011
Vision loss (*n* = 148)	22 (26.5%)	33 (50.8%)	0.002
Hearing loss (*n* = 147)	10 (12.2%)	18 (27.7%)	0.017
Consciousness disturbance (*n* = 148)	9 (10.8%)	13 (20.0%)	0.120
Epilepsy (*n* = 148)	3 (3.6%)	6 (9.2%)	0.182
Limb paralysis (*n* = 147)	7 (8.4%)	0 (0.0%)	0.019
Equilibrium disorder (*n* = 148)	5 (6.0%)	3 (4.6%)	1.0
Speech impairments (*n* = 148)	1 (1.2%)	4 (4.6%)	0.320
Psychiatric symptoms (*n* = 148)	4 (4.8%)	7 (10.8%)	0.213
Pathogen (*n* = 142)			0.223
*C. Neoformans*	4 (5.1%)	7 (10.9%)	
*C. Gattii*	74 (94.9%)	57 (89.1%)	
Hypertension (*n* = 149)	16 (19.0%)	6 (9.2%)	0.094
Diabetes (*n* = 149)	7 (8.3%)	2 (3.1%)	0.300
Hepatitis B (*n* = 149)	6 (7.1%)	9 (13.8%)	0.177
Stroke (*n* = 149)	2 (2.4%)	1 (1.5%)	0.717
Immune disease (*n* = 149)			0.074
Behcet’s disease	1 (1.2%)	0 (0.0%)	
Nephrotic syndrome	6 (7.1%)	0 (0.0%)	
Rheumatoid arthritis	2 (2.4%)	0 (0.0%)	
Myasthenia gravis	1 (1.2%)	0 (0.0%)	
Systemic lupus erythematosus	4 (4.8%)	2 (3.1%)	
Neuromyelitis optica spectrum disorder	0 (0.0%)	1 (1.5%)	
Immunosuppressants (*n* = 148)	16 (19.3%)	3 (4.6%)	0.008

**Table 2:** Treatment regimen.

Item	Control Group (*n* = 84)	PIIRS Group (*n* = 65)	*p* Value
Before admission			
Voriconazole (*n* = 128)	4 (5.6%)	1 (1.8%)	0.380
Flucytosine (*n* = 128)	12 (16.9%)	11 (19.3%)	0.726
Amphotericin B (*n* = 128)	24 (33.8%)	19 (33.3%)	0.955
Fluconazole (*n* = 128)	15 (21.1%)	15 (26.3%)	0.491
Glucocorticoids (*n* = 148)	3 (4.3%)	3 (5.3%)	0.796
After admission			
Voriconazole (*n* = 142)	37 (46.8%)	27 (42.9%)	0.636
Flucytosine (*n* = 146)	80 (97.6%)	63 (98.4%)	1.0
Amphotericin B (*n* = 143)	60 (75.0%)	57 (90.5%)	0.017
Liposomal amphotericin B (*n* = 138)	19 (23.2%)	9 (16.7%)	0.359
Fluconazole (*n* = 145)	51 (63.0%)	40 (62.5%)	0.954
Glucocorticoids were used before or concurrently with antifungal treatment (*n* = 145)	38 (46.9%)	18 (28.1%)	0.021
Ventriculoperitoneal shunt (*n* = 148)	21 (25.0%)	42 (64.6%)	<0.001
From onset to ventriculoperitoneal shunt (d), median [Q1, Q3] (*n* = 65)	49.00 (31.00, 67.00)	48.00 (31.00, 72.00)	1.0
From ventriculoperitoneal shunt to PIIRS (d) (*n* = 42)	-	38.14 ± 49.57	-

**Table 3:** Laboratory data.

Item	Control (*n* = 84)	PIIRS (*n* = 65)	*p* Value
Immunoglobulin G (IgG), g/L, median [Q1, Q3] (*n* = 145)	9.96 (7.91, 11.39)	10.11 (8.70, 11.87)	0.274
Immunoglobulin A (IgA), g/L, median [Q1, Q3] (*n* = 145)	1.67 (1.31, 2.09)	1.88 (1.40, 2.33)	0.213
Immunoglobulin M (IgM), g/L, median [Q1, Q3] (*n* = 149)	0.90 (0.55, 1.35)	0.93 (0.71, 1.37)	0.457
Complement component 3 (C3), g/L (*n* = 145)	1.08 ± 0.39	1.26 ± 0.36	0.005
Complement component 4 (C4), g/L, median [Q1, Q3] (*n* = 145)	0.29 (0.21, 0.38)	0.31 (0.25, 0.43)	0.107
Total complement activity (CH50), U/mL (*n* = 145)	47.95 ± 15.50	49.66 ± 15.20	0.509
Thyroid peroxidase antibody (TPOAb), IU/mL, median [Q1, Q3] (*n* = 125)	23.00 (1.65, 28.00)	18.00 (0.38, 28.00)	0.270
Thyroglobulin antibody (TGAb), IU/mL, median [Q1, Q3] (*n* = 125)	12.85 (1.52, 23.30)	12.00 (1.03, 22.08)	0.765
CD3^+^/CD45^+^, %, (*n* = 46)	68.06 ± 10.16	72.83 ± 10.47	0.147
CD3^+^CD4^+^, % (*n* = 46)	30.29 ± 10.74	31.44 ± 12.01	0.774
CD3^+^CD8^+^, %, median [Q1, Q3] (*n* = 46)	38.50 (32.00, 41.70)	39.80 (24.50, 43.50)	0.558
CD4^+^/CD8^+^, % (*n* = 46)	1.16 ± 0.56	1.09 ± 0.59	0.664
CD3^−^CD19^+^, % (*n* = 46)	12.29 ± 3.43	10.91 ± 3.89	0.229
CD3^−^CD16^+^CD56^+^, %, median [Q1, Q3] (*n* = 46)	15.50 (11.60, 19.00)	17.00 (10.70, 21.00)	0.558
25-Hydroxyvitamin D (25-OH-D), nmol/L, median [Q1, Q3] (*n* = 135)	58.10 (43.00, 74.00)	61.90 (44.00, 68.00)	0.871
Neutrophil count (NEUT#), ×10^9^/L, median [Q1, Q3] (*n* = 149)	6.39 (4.54, 10.04)	7.31 (4.68, 10.34)	0.317
Neutrophils (NEUT%), %, median [Q1, Q3] (*n* = 149)	0.75 (0.66, 0.81)	0.76 (0.67, 0.82)	0.444
C-reactive protein (CRP), mg/L, median [Q1, Q3] (*n* = 149)	4.65 (1.45, 13.80)	8.80 (2.50, 25.00)	0.043
Procalcitonin (PCT), ng/mL, median [Q1, Q3] (*n* = 120)	4.72 (3.83, 5.20)	0.09 (0.05, 0.25)	0.374
Total cholesterol (CHOL), mmol/L, median [Q1, Q3] (*n* = 149)	4.65 (3.81, 5.21)	4.72 (3.83, 5.20)	0.986
Indirect bilirubin (IB), µmol/L, median [Q1, Q3] (*n* = 149)	6.10 (4.10, 8.40)	6.10 (3.80, 8.20)	0.806
Interleukin-6 (IL-6), pg/mL, median [Q1, Q3] (*n* = 56)	5.96 (3.13, 14.26)	9.56 (3.20, 16.39)	0.511
Erythrocyte sedimentation rate (ESR), mm/h, median [Q1, Q3] (*n* = 148)	21.50 (13.00, 38.50)	25.50 (12.00, 44.00)	0.920
D-dimer, mg/L FEU, median [Q1, Q3] (*n* = 119)	25.50 (12.00, 44.00)	0.68 (0.43, 1.57)	0.177
D-glucan, pg/mL, median [Q1, Q3] (*n* = 126)	10.00 (10.00, 17.80)	10.00 (10.00, 21.79)	0.660
Lumbar puncture opening pressure, mmH_2_O, median [Q1, Q3] (*n* = 149)	197.50 (154.00, 275.00)	275.00 (170.00, 330.00)	0.008
Granulocytes (GRAN), median [Q1, Q3] (*n* = 129)	0.20 (0.10, 0.45)	0.20 (0.10, 0.39)	0.682
Monocytes (MONO), median [Q1, Q3] (*n* = 129)	0.80 (0.55, 0.90)	0.80 (0.61, 0.90)	0.981
Cerebrospinal fluid glucose (CSF-GLU), mmol/L (*n* = 149)	2.01 ± 1.19	1.72 ± 1.06	0.121
Cerebrospinal fluid chloride (CSF-Cl), mmol/L, median [Q1, Q3] (*n* = 149)	117.85 (113.00, 122.50)	115.70 (109.00, 118.90)	0.012
Cerebrospinal fluid total protein (CSF-TP), g/L, median [Q1, Q3] (*n* = 149)	0.76 (0.43, 1.15)	0.80 (0.50, 1.15)	0.354
Cerebrospinal fluid adenosine deaminase (CSF-ADA), U/L, median [Q1, Q3] (*n* = 148)	0.00 (0.00, 1.00)	0.00 (0.00, 2.00)	0.423
Smear (*n* = 149), median [Q1, Q3]	469.50 (10.00, 4154.00)	5376.00 (514.00, 23,168.00)	<0.001

**Table 4:** Multivariable analysis.

Item	Odds Ratio [95% CI]	*p* Value
Vomiting	2.12 [0.88, 5.26]	0.099
Vision change	2.59 [1.03, 6.71]	0.056
Hearing change	1.25 [0.35, 4.41]	0.722
Immunosuppressant before PIIRS	0.84 [0.14, 4.17]	0.836
Amphotericin B	1.52 [0.45, 5.64]	0.514
Glucocorticoids were used before or concurrently with antifungal treatment	0.43 [0.16, 1.10]	0.084
Ventriculoperitoneal shunt	4.64 [1.84, 12.46]	0.001
Complement C3	5.78 [1.55, 24.42]	0.012
C-reactive protein	1.00 [0.98, 1.01]	0.750
Lumbar puncture opening pressure	1.01 [1.00, 1.01]	0.008
Cerebrospinal fluid chloride	1.02 [0.97, 1.06]	0.303
Smear	1.00 [1.00, 1.00]	0.793

**Table 5:** Performance of classification models.

Model	AUC (95% CI)	Accuracy	Sensitivity	Specificity	F1 Score
Test set					
ROI only	0.877 (0.714–0.991)	0.840	0.818	0.857	0.818
ROI + location	0.822 (0.800–1.000) *	0.840	1.000	0.714	0.846
ROI + C3	0.649 (0.429–0.864) *	0.680	0.818	0.571	0.692
ROI + location + C3	0.948 (0.840–1.000) *	0.880	1.000	0.786	0.880
Validation set					
ROI only	0.875 (0.656–1.000)	0.789	1.000	0.636	0.800
ROI + location	0.920 (0.733–1.000)	0.947	0.875	1.000	0.933
ROI + C3	0.739 (0.411–0.982)	0.789	0.750	0.818	0.750
ROI + location + C3	0.943 (0.814–1.000)	0.895	0.750	1.000	0.857

* Pairwise DeLong Test: ROI + C3 vs. ROI + location, *p* = 0.0436; ROI + C3 vs. ROI + location + C3, *p* = 0.0137.

## Data Availability

The original contributions presented in this study are included in the article/Supplementary Materials. Further inquiries can be directed to the corresponding author.

## Supplementary Materials

The following supporting information can be downloaded at https://www.mdpi.com/article/10.3390/diagnostics16152370/s1: Supplementary Table S1: First-order statistics; Supplementary Table S2: Shape features; Supplementary Table S3: Gray-level co-occurrence matrix (GLCM) texture features; Supplementary Table S4: Gray-level run length matrix (GLRLM) features; Supplementary Table S5: Gray-level size zone matrix (GLSZM) features; Supplementary Table S6: Neighborhood gray-tone difference matrix (NGTDM) features; Supplementary Table S7: Wavelet features; Supplementary Figure S1: Model performance (3-fold cross-validation).

## Abbreviations

The following abbreviations are used in this manuscript:

AdamW	Adaptive moment estimation with weight decay
ReLU	Rectified linear unit
C3	Complement component 3
FLAIR	Fluid-attenuated inversion recovery
BCE	Binary cross-entropy
CI	Confidence interval
SD	Standard deviation
MLP	Multi-layer perceptron
Dice	Dice similarity coefficient
GLCM	Gray-level co-occurrence matrix
GLRLM	Gray-level run length matrix
GLSZM	Gray-level size zone matrix
NGTDM	Neighborhood gray-tone difference matrix
IoU	Intersection over union
AUC	Area under the curve
CM	Cryptococcal meningitis
CNS	Central nervous system
CRP	C-reactive protein
CSF	Cerebrospinal fluid
ESR	Erythrocyte sedimentation rate
IL-6	Interleukin-6
LIME	Local Interpretable Model-agnostic Explanations
MRI	Magnetic resonance imaging
PIIRS	Post-infectious inflammatory response syndrome
SHAP	SHapley Additive exPlanations
VPS	Ventriculoperitoneal shunt
ICU	Intensive care unit
