# Supplementary material for: Prediction of Post-Infectious Inflammatory Response Syndrome in Patients with Cryptococcal Meningitis Based on Clinical and Radiomics Data
Source: Diagnostics (Basel). 2026 Jul 28;16(15):2370. doi: 10.3390/diagnostics16152370 (PMC13464751; doi:10.3390/diagnostics16152370)

Supplementary Table S1 First-order statistics

Item	Control group (n = 69)	PIIRS group (n = 53)	P value
Energy	134.70 ± 267.54	101.28 ± 147.21	0.320
Entropy	-773.30 ± 847.20	-1424.76 ± 2008.17	**0.028**
Kurtosis	1.57 ± 1.87	3.41 ± 6.73	0.236
Mean	0.62 ± 0.12	0.70 ± 0.16	**0.009**
Root mean square (RMS)	0.63 ± 0.12	0.71 ± 0.16	**0.011**
Skewness	-0.91 ± 0.51	-1.27 ± 0.99	0.078
Total energy	134.70 ± 267.54	101.28 ± 147.21	0.320
Uniformity	21247.80 ± 57700.57	116355.65 ± 451205.93	**0.024**
Max ROI area	15588.58 ± 51850.71	4011.32 ± 9825.92	0.071
Mean ROI area	2609.96 ± 8225.90	1332.10 ± 3028.70	0.131
Number of ROI slices	14.28 ± 3.64	12.00 ± 4.21	**0.002**
Total ROI area	40320.91 ± 133926.26	19741.70 ± 46700.24	**0.049**

Supplementary Table S2 Shape features

Item	Control group (n = 69)	PIIRS group (n = 53)	P value
Area	149.76 ± 527.24	65.50 ± 191.29	0.128
Aspect ratio	0.33 ± 0.11	0.30 ± 0.11	0.108
Circularity	0.24 ± 0.07	0.25 ± 0.08	0.524
Compactness	7.67 ± 4.51	6.81 ± 3.68	0.100
Convexity	0.85 ± 0.08	0.87 ± 0.09	0.110
Eccentricity	0.78 ± 0.11	0.73 ± 0.19	0.224
Elongation	0.55 ± 0.09	0.51 ± 0.14	0.236
Equivalent diameter	6.65 ± 7.84	5.57 ± 5.60	0.150
Extent	0.23 ± 0.06	0.22 ± 0.06	0.190
Perimeter	63.34 ± 109.58	53.03 ± 81.39	0.143
Solidity	0.55 ± 0.10	0.55 ± 0.11	0.568

Supplementary Table S3 Gray level co-occurrence matrix (GLCM) texture features

Item	Control group (n = 69)	PIIRS group (n = 53)	P value
Angular second moment (distance 1)	0.75 ± 0.15	0.73 ± 0.15	0.182

Angular second moment (distance 2)	0.74 ± 0.15	0.72 ± 0.15	0.156
Angular second moment (distance 3)	0.73 ± 0.15	0.69 ± 0.16	0.127
Contrast (distance 1)	154.10 ± 177.68	277.80 ± 304.60	**0.010**
Contrast (distance 2)	161.37 ± 160.06	274.27 ± 264.78	**0.005**
Contrast (distance 3)	151.49 ± 100.02	213.65 ± 152.34	**0.009**
Correlation (distance 1)	0.40 ± 0.14	0.37 ± 0.18	0.362
Correlation (distance 2)	0.32 ± 0.11	0.30 ± 0.14	0.357
Correlation (distance 3)	0.23 ± 0.10	0.25 ± 0.12	0.500
Dissimilarity (distance 1)	3.94 ± 3.54	5.83 ± 5.49	**0.049**
Dissimilarity (distance 2)	4.06 ± 3.12	5.75 ± 4.78	**0.019**
Dissimilarity (distance 3)	3.72 ± 1.93	4.52 ± 2.48	**0.044**
Energy (distance 1)	0.83 ± 0.12	0.82 ± 0.11	0.315
Energy (distance 2)	0.82 ± 0.12	0.81 ± 0.12	0.242
Energy (distance 3)	0.81 ± 0.14	0.77 ± 0.16	0.162
Homogeneity (distance 1)	0.83 ± 0.14	0.80 ± 0.16	0.530
Homogeneity (distance 2)	0.82 ± 0.15	0.79 ± 0.16	0.379
Homogeneity (distance 3)	0.80 ± 0.15	0.77 ± 0.17	0.203

Supplementary Table S4 Gray level run length matrix (GLRLM) features

Item	Control group (n = 69)	PIIRS group (n = 53)	P value
Gray level non-uniformity	97.25 ± 58.52	85.38 ± 61.60	0.130
High gray level run emphasis	823.95 ± 342.50	1017.93 ± 442.34	**0.011**
Low gray level run emphasis	0.52 ± 0.08	0.53 ± 0.09	0.640
Long run emphasis	615.55 ± 412.10	552.80 ± 395.38	0.396
Long run high gray level emphasis	1860.65 ± 1472.99	2724.45 ± 3517.18	0.056

Long run low gray level emphasis	614.94 ± 412.16	552.01 ± 395.46	0.396
Run length non-uniformity	204.23 ± 510.80	116.81 ± 204.65	**0.027**
Run percentage	0.22 ± 0.16	0.26 ± 0.19	0.343
Short run emphasis	0.55 ± 0.10	0.55 ± 0.13	0.810
Short run high gray level emphasis	781.61 ± 306.28	924.84 ± 408.73	**0.040**
Short run low gray level emphasis	0.08 ± 0.04	0.10 ± 0.07	0.308

Supplementary Table S5 Gray level size zone matrix (GLSZM) features

Item	Control group (n = 69)	PIIRS group (n = 53)	P value
Gray level non-uniformity	11.04 ± 13.95	10.04 ± 16.03	0.145
High gray level zone emphasis	1673.43 ± 618.16	2078.37 ± 828.18	**0.008**
Large area emphasis	150540.87 ± 1250404.43	54.35 ± 231.34	0.123
Large area high gray level emphasis	186531.40 ± 1258057.94	219166.93 ± 948128.03	**0.049**
Large area low gray level emphasis	150531.14 ± 1250405.43	0.01 ± 0.06	0.648
Low gray level zone emphasis	0.00 ± 0.01	0.00 ± 0.00	**0.005**
Small area emphasis	0.90 ± 0.03	0.85 ± 0.10	0.085
Small area high gray level emphasis	1467.90 ± 510.99	1663.52 ± 580.95	0.060
Small area low gray level emphasis	0.00 ± 0.00	0.00 ± 0.00	**0.005**
Zone percentage	0.83 ± 0.08	0.76 ± 0.18	0.108
Zone size non-uniformity	189.05 ± 426.52	103.82 ± 154.57	**0.022**

Supplementary Table S6 Neighborhood gray tone difference matrix (NGTDM) features

Item	Control group (n = 69)	PIIRS group (n = 53)	P value
Busyness	215744400.60 ± 1792107585.71	18374153362.46 ± 88796812525.79	**0.006**

Coarseness	7627765.12 ± 63360975.31	0.07 ± 0.06	0.786	
Complexity	2541.46 ± 2153.49	2025.94 ± 2041.70	**0.015**	
Contrast	9.40 ± 45.44	14.84 ± 36.63	0.240	
Strength	21.56 ± 7.55	19.56 ± 8.15	0.167	

Supplementary Table S7 Wavelet features

Item	Control group (n = 69)	PIIRS group (n = 53)	P value
Level 1 high-high energy	1.35 ± 1.63	1.12 ± 1.30	0.303
Level 1 high-high entropy	-864.12 ± 1451.84	-787.74 ± 818.47	0.716
Level 1 high-high mean	0.00 ± 0.00	-0.00 ± 0.00	0.053
Level 1 high-low energy	1.77 ± 2.20	1.47 ± 1.81	0.259
Level 1 high-low entropy	-743.25 ± 1448.56	-666.59 ± 1091.39	0.443
Level 1 high-low mean	-0.00 ± 0.00	-0.00 ± 0.00	0.818
Level 1 low-high energy	4.74 ± 5.62	3.99 ± 4.42	0.266
Level 1 low-high entropy	-314.42 ± 111.35	-366.02 ± 335.96	0.600
Level 1 low-high mean	-0.00 ± 0.00	0.00 ± 0.00	0.507
Level 1 low-low energy	25.82 ± 59.48	18.74 ± 30.21	0.328
Level 1 low-low entropy	-330.08 ± 126.13	-323.53 ± 148.67	0.490
Level 1 low-low mean	0.00 ± 0.01	0.00 ± 0.00	0.203
Level 2 high-high energy	0.24 ± 0.31	0.21 ± 0.26	0.367
Level 2 high-high entropy	-2260.22 ± 6366.68	-1694.33 ± 2487.52	0.478
Level 2 high-high mean	-0.00 ± 0.00	-0.00 ± 0.00	0.136
Level 2 high-low energy	0.36 ± 0.51	0.31 ± 0.41	0.300
Level 2 high-low entropy	-2153.00 ± 6428.74	-1973.71 ± 3689.54	0.544
Level 2 high-low mean	-0.00 ± 0.00	-0.00 ± 0.00	0.381
Level 2 low-high energy	0.91 ± 1.23	0.80 ± 0.95	0.393
Level 2 low-high entropy	-915.02 ± 1423.52	-849.93 ± 937.50	0.640
Level 2 low-high mean	-0.00 ± 0.00	0.00 ± 0.00	0.086

Supplementary Figure S1 Model performance (3-fold cross-validation)

Test set

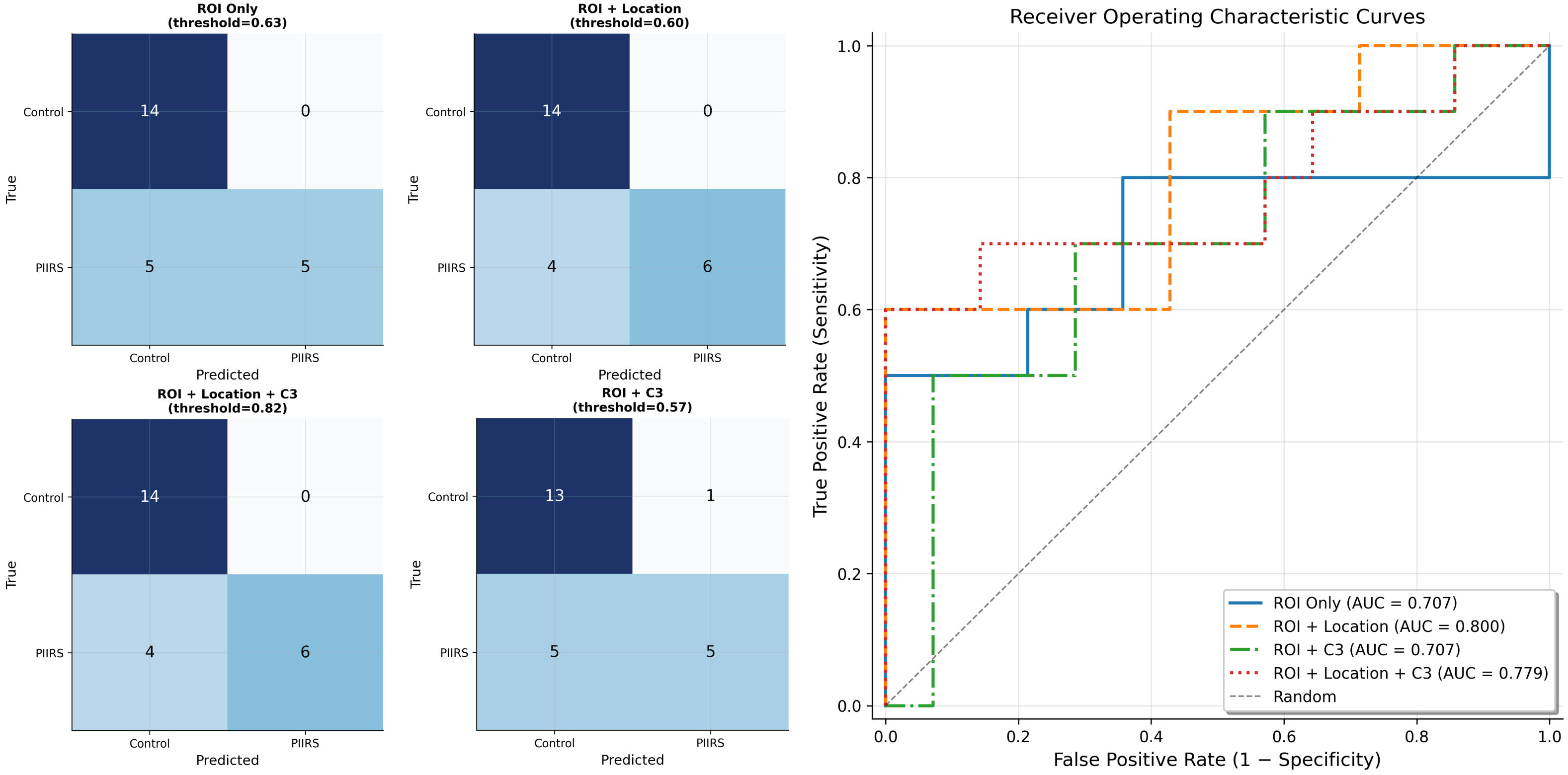

Validation set

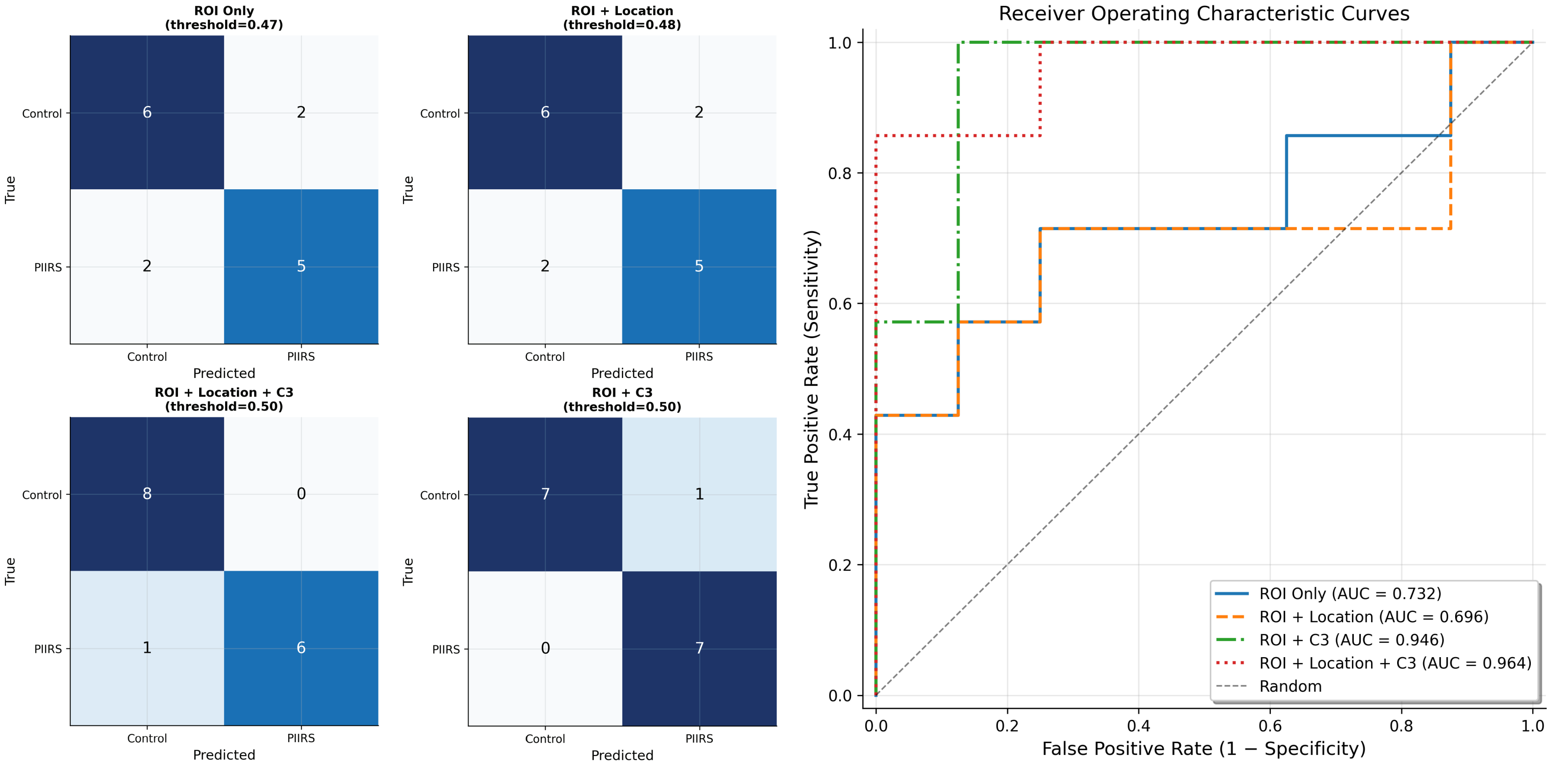

Supplement: Supplementary file 1 [file diagnostics-16-02370-s001.zip › diagnostics-4310163-supplementary.pdf]
